# Quantifying the efficacy of checkpoint inhibitors on CD8^+^ cytotoxic T cells for immunotherapeutic applications via single-cell interaction

**DOI:** 10.1038/s41419-020-03173-7

**Published:** 2020-11-13

**Authors:** Matthew Ryan Sullivan, Giovanni Stefano Ugolini, Saheli Sarkar, Wenjing Kang, Evan Carlton Smith, Seamus Mckenney, Tania Konry

**Affiliations:** Department of Pharmaceutical Sciences, School of Pharmacy, Bouve College of Health Sciences, Northeastern University, 140 The Fenway, Boston, MA 02115 USA

**Keywords:** Cancer models, Cellular imaging, Phenotypic screening, Immunotherapy, Cancer immunotherapy

## Abstract

The inhibition of the PD1/PDL1 pathway has led to remarkable clinical success for cancer treatment in some patients. Many, however, exhibit little to no response to this treatment. To increase the efficacy of PD1 inhibition, additional checkpoint inhibitors are being explored as combination therapy options. TSR-042 and TSR-033 are novel antibodies for the inhibition of the PD1 and LAG3 pathways, respectively, and are intended for combination therapy. Here, we explore the effect on cellular interactions of TSR-042 and TSR-033 alone and in combination at the single-cell level. Utilizing our droplet microfluidic platform, we use time-lapse microscopy to observe the effects of these antibodies on calcium flux in CD8^+^ T cells upon antigen presentation, as well as their effect on the cytotoxic potential of CD8^+^ T cells on human breast cancer cells. This platform allowed us to investigate the interactions between these treatments and their impacts on T-cell activity in greater detail than previously applied in vitro tests. The novel parameters we were able to observe included effects on the exact time to target cell killing, contact times, and potential for serial-killing by CD8^+^ T cells. We found that inhibition of LAG3 with TSR-033 resulted in a significant increase in calcium fluctuations of CD8^+^ T cells in contact with dendritic cells. We also found that the combination of TSR-042 and TSR-033 appears to synergistically increase tumor cell killing and the single-cell level. This study provides a novel single-cell-based assessment of the impact these checkpoint inhibitors have on cellular interactions with CD8^+^ T cells.

## Introduction

The checkpoint pathway is an integral component of the immune system, maintaining self-tolerance and preventing unnecessary inflammation and cytotoxicity. It also presents a mechanism of immune evasion utilized by many cancers via high expression of checkpoint ligands, inducing exhaustion and anergy in cytotoxic immune cells that might otherwise recognize and kill the tumor cells^1^. Programmed cell death protein-1 (PD1) is currently the most well-characterized and documented checkpoint receptor. PD1 and/or PDL1 inhibition has resulted in remarkable clinical success in some patients, however, only a limited subset successfully responds to PD1 treatment alone. The remainder of patients experiences little to no beneficial health outcomes^2,3^. Numerous potential pathways have been identified that should produce a synergistic effect with PD-1 inhibition^4,5^. The lymphocyte-activation gene 3 (LAG3) receptor facilitates an additional checkpoint pathway, and is commonly co-expressed with PD1 on T cells, and presents a major target of interest for combination therapy^3,6–10^. Two novel antibodies were previously developed for the goal of combination therapy: TSR-042, an anti-PD1 antibody, and TSR-033, an anti-LAG3 antibody. Early pre-clinical trials have shown promising efficacy for the therapeutic potential of these two antibodies in tumor reduction in vivo in rats and increasing CD8^+^ T-cell secretory activity in vitro^11^. It is unclear, however, if the tumor-killing observed in rats will be matched in humans.

There is widespread consensus that improved pre-clinical models could lead to higher predictive potential and better therapies in clinical trials. Animal models often lead to confounding species-specific results, and in vitro tests for immunotherapies are often aimed at quantifying secondary outcomes such as cytokine release, bulk cell proliferation or cytotoxicity, and surface marker analysis^10–12^. Most in vitro tests cannot sensitively observe tumor cell killing and immune cell activation. Additionally, subpopulations of cells will display trends that can be lost in bulk cell culture utilized in standard in vitro methods^13^. To overcome this, cell pairing at the single-cell level provides a promising strategy^14–18^. Pairing target and effector cells in independent environments indeed give the chance to detect functional outcomes and kinetics (e.g., cell-cell contact, cell killing, etc.). We have previously described a single-cell droplet-based microfluidic platform allowing capture and time-lapse imaging of single cells or cell pairs to observe parameters such as cancer cell killing and synaptic contact between cells^19–21^.

We here describe the use of our droplet-based single-cell platform assay to test the effects of TSR-042 and TSR-033 on several aspects of T lymphocyte anti-tumor functions. We analyzed calcium signaling of CD8^+^ T cells paired to dendritic cells to determine the antibody’s effect on immune communication and T-cell activation. Additionally, we tested the impact of TSR-042, TSR-033, and their combination on CD8^+^ T-cell cytotoxicity and interactions with cancer cells by co-encapsulation of T cells with two cell lines of interest. MDA-MB-231 adenocarcinoma cells, a triple-negative breast cancer (TNBC) cell line, and SKOV3, and ovarian cancer cell line, were selected as both TNBC and ovarian cancer are targets of interest for checkpoint inhibitor therapy but have shown inadequate clinical success with PD1/PDL1 inhibition alone^22,23^. To compare the predictivity of our advanced model to a standard in vitro model, additional cytotoxicity experiments were performed using a commercial 96 well LDH release assay. Our results displayed an increase in calcium peak frequency in anti-LAG3-treated T cells. We also observed a significant increase in the cytotoxicity of T cells towards TNBC cells with anti-PD1 treatment that was further enhanced with the co-administration of antibodies, but a lack of increase in cytotoxicity towards ovarian cancer cells. Importantly, this result did not emerge from standard in vitro assays. These findings support the potential of these antibodies as combination immunotherapy and highlight the potential of advanced assays for providing enabling insights in pre-clinical testing of immunotherapies. To our knowledge, this is the first report on the effects of these checkpoint inhibitors at the single-cell level, providing a greater understanding of their impact on the cellular interactions of CD8^+^ T cells.

## Methods

### Microfluidic device fabrication and loading

**Fig. 1:**
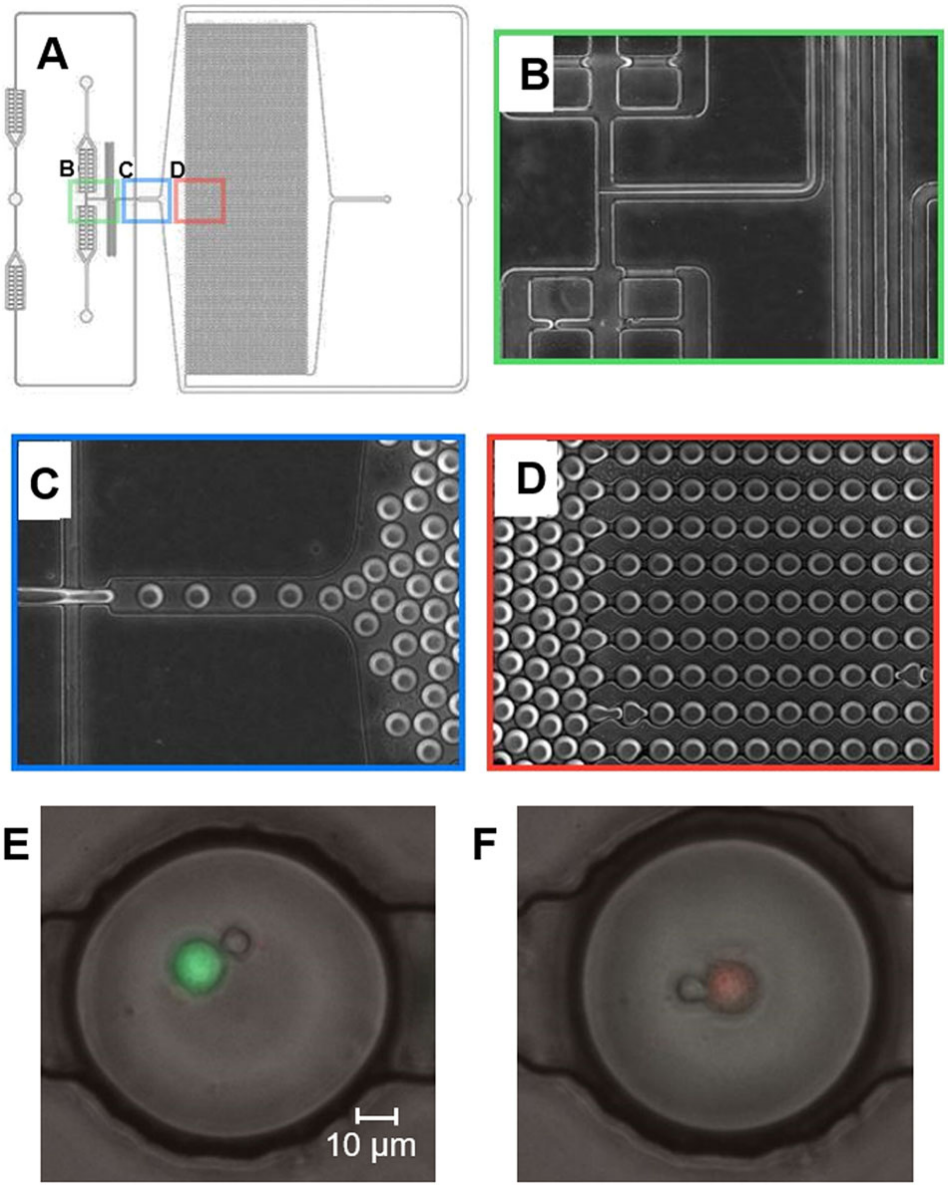
Microfluidic Device Design and Microscope Images. **A** Schematic of 4000 docking-site droplet-based microfluidic device. **B** Aqueous inlets and cell mixing region. **C** Droplet forming junction. **D** Droplet docking array. **E**, **F** Representative images of CD8^+^ T cell and cancer cell co-encapsulations with live cancer cells labeled green (**E**) and dead cancer cells labeled red (**F**).

Microfluidic devices (shown in Fig. 1) were fabricated by standard replica molding of polydimethylsiloxane (PDMS) (10:1 mixing ratio, 1 h curing at 80 °C) on microstructured wafers used as molds. The resulting PDMS layers are then punched with 0.75 mm biopsy punchers to provide channel inlets/outlets and bonded to glass slides by plasma exposure. We employed microfluidic devices design previously described^21,24^. The design features a T-shaped droplet generator, generating aqueous-in-oil droplets (Fig. 1C). The aqueous phase is composed of media containing each respective cell type (T cells and cancer cells) and introduced via a serpentine channel from two separate inlets so that different cell types come into contact only at droplet generation. Oil and media are flowed continuously into the device at controlled rates (~4:1 oil:aqueous phase ratio) using syringe pumps (Harvard Apparatus, Holliston, MA). Downstream to the droplet generator the microfluidic devices feature an array with 4000 droplet docking sites where droplets are trapped for subsequent time-lapse imaging steps. The size of the droplets can be controlled by adjusting flow rates, with an ideal droplet size of ~90 µm in diameter. This design has been previously established by our lab as an effective means of co-encapsulating cell pairs to observe interactions in past works.

### Cell culture and activation

CD8^+^ T cells were purchased from STEMCELL Technologies (Cambridge, MA). T-cell activation was performed by incubating cells with anti-CD3/CD28 DynaBeads (ThermoFisher Scientific, Waltham, MA) at a 1:1 cell to bead ratio with 30 U/mL IL-2 in RPMI 1640 complete media with 10% FBS and 1% Antibiotic-Antimycotic (Biological Industries, Cromwell, CT) for 6 days. T cells were rested overnight in fresh media without the presence of DynaBeads or IL-2 before all experiments. Following this one-week, activation, T cells were incubated with checkpoint inhibitor antibodies (TSR-042 and TSR-033) for 2 h prior to the experiment. Checkpoint inhibitor antibodies were provided by TESARO and used at concentrations of 30 µg/mL for all experiments. Control T cells were given the same 1-week activation but did not receive antibody treatment. DCs (StemCell Technologies) were activated for 24 h in 200 ng/mL Lipopolysaccharides (LPS) (Sigma Aldrich, St. Louis, MO). Before experiments, DCs were incubated with the superantigen Staphylococcus Enterotoxin B (SEB) (Fisher Scientific, Hampton, NH) for 2 h. SEB served as a non-specific antigen to activated T cells when presented by DCs. MDA-MB-231 cells and SKOV3 cells were acquired via ATCC (Manassas, VA). MDA-MB-231 were grown in DMEM with 1 g/L glucose (Corning, Manassas, VA) with 10% FBS and 1% Antibiotic-Antimycotic. SKOV3 were grown in RPMI 1640 complete media with 10% FBS and 1% Antibiotic-Antimycotic (Biological Industries, Cromwell, CT).

### Imaging and cell analysis

The procedures used for analysis and microscopic imaging are described in the Supplementary Methods.

### Standard in vitro cytotoxicity

The Promega Cytotox 96 kit has been utilized frequently as a cytotoxicity assay for immune cells and was chosen as a standard comparison^25–27^. The protocol is described in detail in the Supplementary Methods.

### Flow cytometry

The procedures used for flow cytometry analysis are described in the Supplementary Methods.

## Results

### Checkpoint receptor expression on CD8^+^ T cells and ligand expression of MDA-MB-231 breast cancer cells

**Fig. 2:**
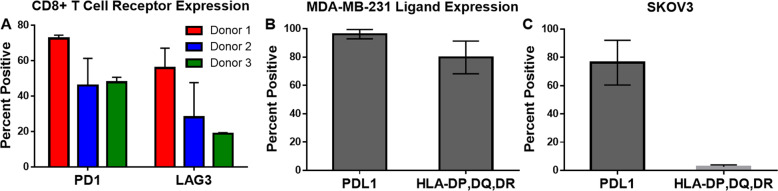
Flow cytometry analysis of target and effector cell surface markers. **A** Checkpoint receptor expression of CD8^+^ T cells for multiple donors. The number of repeats for each donor are donor 1: 3, donor 2: 5, and donor 3: 2. **B** Checkpoint ligand expression on MDA-MB-231 cells (*n* = 2). **C** Checkpoint ligand expression of SKOV3 cells (*n* = 2).

To better understand the extent to which TSR-042 and TSR-033 may interact with CD8^+^ T cell and the target cells used in these experiments, checkpoint receptor and ligand expression were assessed by flow cytometry (Fig. 2). PD1 and LAG3 receptor expression were measured on the CD8^+^ T cells, while PDL1 (a PD1 ligand) and HLA-DP, DQ, and DR (LAG3 ligands) were chosen as the target ligands on cancer cells.

CD8^+^ T-cell receptor expression varied depending on the donor source (Fig. 2A), particularly with respect to the expression of LAG3. PD1 expression averaged ~70% for the first donor and 50% positive expression for donors two and three. LAG3 expression averaged ~55% for the first donor, 30% for the second, and 20% for the third donor. Figure 2B displays the ligand expression on MDA-MB-231 cell, which had an average of 96% positive for PDL1 expression and 80% positive for HLA-DP, DQ, DR expression. SKOV3 presented a 76% positive expression of PDL1 and only 2.6% positive for HLA-DP, DQ, DR.

### Effects of PD1 and LAG3 inhibition on dendritic cell-induced calcium release of CD8^+^ T cells

**Fig. 3:**
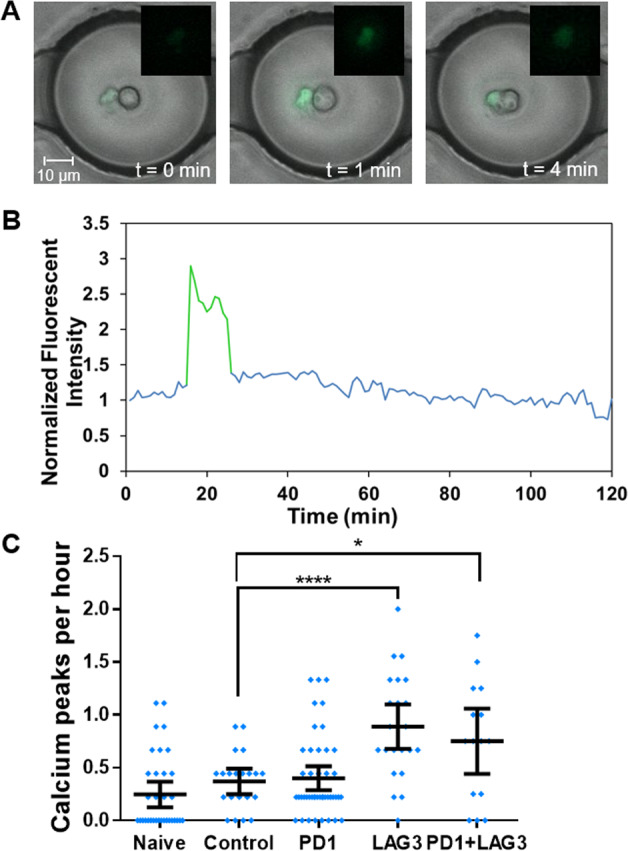
Calcium levels of CD8+ T cells co-encaspulated with DCs. **A** Overlay of fluorescent and merged phase images of a CD8^+^ T-cell co-encapsulated with a dendritic cell before and during a calcium peak. CD^+^ T cells are fluorescent and dendritic cells are unlabeled. **B** Representative normalized fluorescent intensity profile of a single cell displaying with calcium peak highlighted in green. **C** Average calcium peaks for each CD8^+^ T cells over 4.5 h. The sample sizes (*n*) for this experiment are: naive: 35, partially activated control: 21, PD1: 45, LAG3: 24, and PD1^+^ LAG3: 15 cells.

To determine the effect of TSR-042 and TSR-033 on CD8^+^ T-cell sensitivity to activation, we utilized dendritic cells as an antigen-presenting cell and observed calcium fluctuations of CD8^+^ T cells at a single-cell level. T cells were labeled with Fluo-4 NW Calcium Assay dye (Life Technologies, Carlsbad, CA) and paired with dendritic cells in the droplets at a 1:1 ratio (Fig. 3A).

Time-lapse imaging was conducted at for 4.5 h and changes in CD8^+^ T-cell fluorescence were measured for all cells. Figure 3B displays a representative fluorescent intensity profile over time that was generated for a single cell. Calcium peaks with an increase in normalized fluorescent intensity of 0.5 or greater were counted, and each cell’s average Ca^2+^ peaks per hour were recorded (Fig. 3C). Naive CD8^+^ T cells were tested for activity 24 h after thawing with no activation steps. These cells averaged 0.25 Ca^2+^ peaks per hour, with more than half of observed cells not producing any Ca^2+^ peaks. CD8^+^ T cells that had been activated in culture (see “Methods” section) and had no antibody administered were used as the control population. Over 80% of control T cells produced at least 1 Ca^2+^ peak, however, the increase in average peaks per hour to 0.37 was not statistically significant compared to naive T cells. The anti-PD1-treated T cells increased compared to the untreated control, although not significantly, to 0.40 average peaks per hour. Anti-LAG3-treated CD8^+^ T cells did have a statistically significant increase in Ca^2+^ peaks, with an average of 0.89 peaks per hour (*p* < 0.0001). The combination treatment exhibited similar Ca^2+^ signaling to LAG3, with an average of 0.75 peaks per hour.

### PD1 and LAG3 inhibition effects on cytotoxicity and contact time of CD8^+^ T cells

**Fig. 4:**
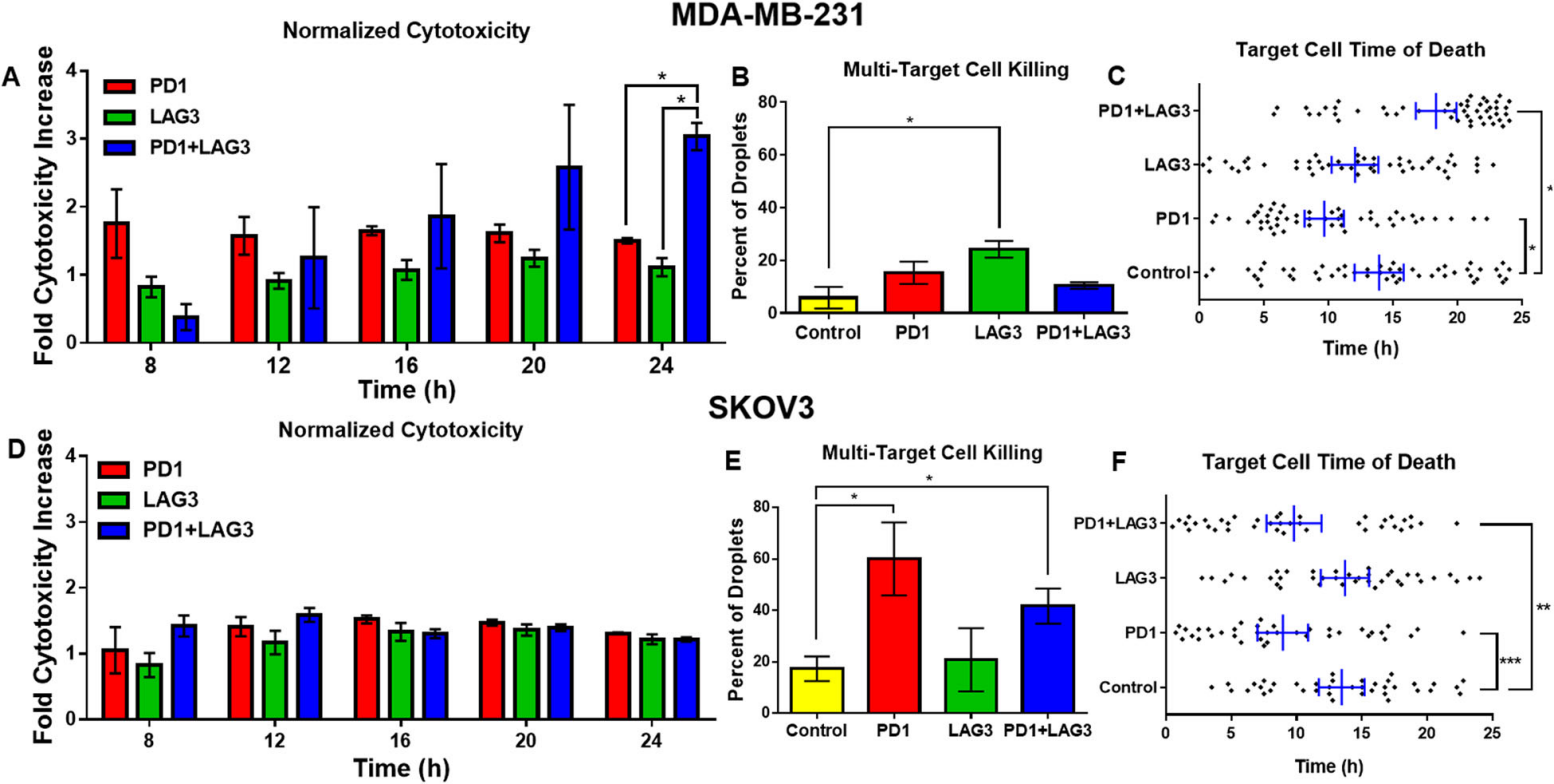
Cytotoxicity results of MDA-MB-231 breast cancer cell SKOV3 ovarian cancer cell co-encapsulations with CD8^+^ T cells. **A**–**C** Target cell is MDA-MB-231. **A** Cytotoxicity of each treatment at multiple timepoints normalized to control. The number of repeats (*n*) for each condition is PD1: 4, LAG3: 4, PD1^+^ LAG3: 3. Error bars represent the standard error from the mean. **B** Serial-killing potential of CD8^+^ T cells paired with two target cells. Events (droplets) observed for each condition are: Control: 37, PD1: 35, LAG3: 24, PD1^+^ LAG3: 32. **C** Target cell time of death analysis for 50 randomly selected cells from droplet co-encapsulations each treatment condition. **D**–**F** Target cell is SKOV3. **D** Normalized cytotoxicity over multiple timepoints normalized to control. The number of repeats (*n*) for each condition is PD1: 4, LAG3: 4, PD1^+^ LAG3: 3. **E** Serial-killing potential. Events (droplets observed of each condition) are: Control: 25, PD1: 10, LAG3: 25, PD1^+^ LAG3: 12. **F** Time of death analysis of 40 randomly selected cells. Error bars represent a 95% confidence interval of the mean.

To assess the effect of TSR-042 and TSR-033 on CD8^+^ T-cell tumor-killing potential at the single-cell level, we co-encapsulated the T cells with target cancer cells in droplets and observed cell interactions for 24 h. Death of the cancer cells and the proportion of time in contact with the T cells were both recorded for cell pairs subject to control conditions and to checkpoint inhibitors alone or in combination. Figure 4A, D displays the cytotoxicity of each condition at 4-h intervals starting at 8 h, normalized by the percent killing by untreated control CD8^+^ T cells from the same donor. Minimal levels of target cell killing were observed before 8 h. For MDA-MB-231, PD1 inhibition resulted in the greatest normalized increase in cytotoxicity at 8 h, while the combination treatment cytotoxicity was lower than control at this time point (Fig. 4A). The combination treatment however continued increasing in cytotoxicity over time, with a fold increase in killing compared to control of about 3 at 24 h, while PD1 inhibition remained constant at about 1.6- to 1.8-fold increase. Cytotoxicity with LAG3 inhibition alone remained relatively similar to control at each time point (fold change of ~1). For SKOV3, the PD1^+^ LAG3 inhibited CD8^+^ T cells had the highest increase in killing at the 8 and 12-h timepoints, however, the treatment conditions leveled off at later timepoints, and no condition exhibited significantly higher cytotoxicity.

To observe the effects of each treatment condition on the ability of CD8^+^ T cells to kill multiple target cells, we performed specific experiments aimed at achieving droplet occupancy of one effector cell and two target cells (see “Methods” section). Subsequently, a droplet containing two tumor cells and a single CD8^+^ T cell were monitored for interactions over 24 h and the percent of droplets with both target cells killed were recorded (Fig. 4B). Unexpectedly, only LAG3 inhibition had a significantly higher percent of droplets with both target cells killed than control (*p* = 0.002). Additionally, only PD1 inhibition had significantly fewer droplets with no target cells killed than control (*p* = 0.003). Surprisingly, the combination treatment did not have greater killing than individual antibodies. To provide additional insight into this aspect, we analyzed a random sampling of 50 cells from the 1:1 effector-target results for each condition and compared the MDA-MB-231 cell times of death (Fig. 4C). The combination treatment indeed led to a higher time-to-death of target cells compared to the other conditions (averaging 18.3 h), while the PD1 inhibition alone condition had the fastest average killing time (averaging 9.6 h). With SKOV3, the PD1 inhibited and combination conditions had the highest rates of multi-cell killing (*p* < 0.05, Fig. 4E). This again matched the time-to-death data (Fig. 4F), where the PD1 inhibited and combination conditions had a significantly less average time to killing target cells than control (*p* = 0.008 and *p* = 0.084, respectively).

**Fig. 5:**
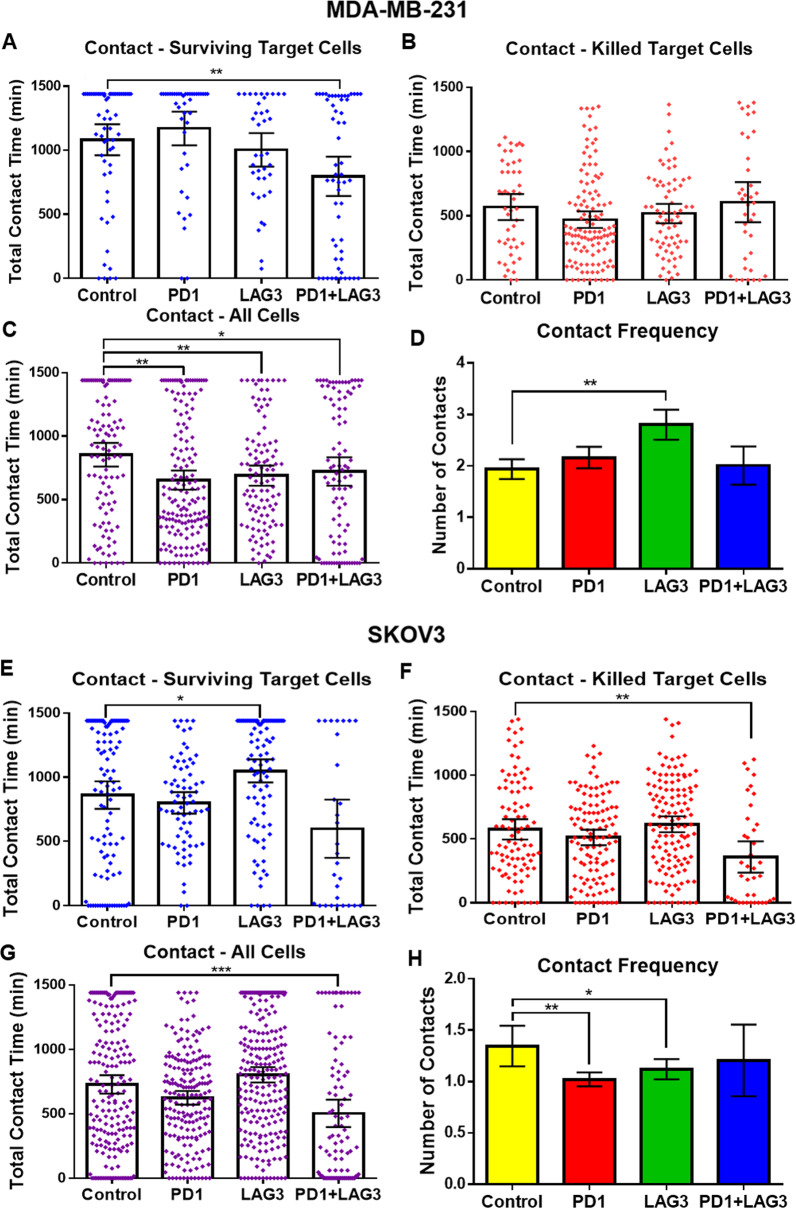
Contact results of MDA-MB-231 and SKOV3 co-encapsulations with CD8^+^ T cells. **A**–**D** Target cell is MDA-MB-231. **A** Total contact time between CD8^+^ T cells and target cells which survived 24 h in droplets. The total number of cells observed for each condition is Control: 60, PD1: 42, LAG3: 38, PD1^++^ LAG3: 57. **B** Total contact time between CD8^+^ T cells and target cells that in target cells died during imaging. The total number of cells observed for each condition is Control: 48, PD1: 118, LAG3: 70, PD1^+^ LAG3: 37. **C** Total contact time for all CD8^+^ T and target cell co-encapsulations (live and dead combined). **D** Average frequency of contact during 24-h experiments combined. *N* = Control: 327, PD1: 375, LAG3: 235, PD1^+^ LAG3: 142. **E**–**H** Target cell is SKOV3. **E** Total contact time between CD8^+^ T and target cells which survived 24 h in droplets. *N* = Control: 105, PD1: 72, LAG3: 96, PD1^+^ LAG3: 29. **F** Total contact time between CD8^+^ T cells and target cells that in target cells died during imaging. *N* = Control: 92, PD1: 113, LAG3: 127, PD1^+^ LAG3: 38. **G** Total contact time for all CD8^+^ T and target cell co-encapsulations (live and dead combined). *N* = Control: 197, PD1: 185, LAG3: 223, PD1^+^ LAG3: 96. **H** Average frequency of contact during 24-h experiments combined. *N* = Control: 197, PD1: 185, LAG3: 223, PD1^+^ LAG3: 96. Error bars represent the 95% confidence interval contact time for all conditions.

The droplet-based single-cell imaging approach also allowed us to observe the length of synaptic contact between individual T cells and cancer cells for each treatment condition. Data from all experimental repeats were binned based on target cell death. In co-encapsulations where MDA-MB-231 cells were not killed (Fig. 5A), combination treatment displayed a significantly lower average contact time compared to control (*p*-value = 0.0016). When combining the data from all conditions (Fig. 5C), all treatment conditions have significantly lower average contact times than control (*p* < 0.05). In co-encapsulations of CD8^+^ T cells with SKOV3, the combination of both antibodies produced a significant decrease in contact time in the killed (Fig. 5F) and combined (Fig. 5G) data, but not with surviving target cells (Fig. 5E). LAG3 inhibition produced a significant increase in the contact time of CD8^+^ T cells with SKOV3 (*p* = 0.018).

### Cytotoxicity comparison with 96 well standard assay

**Fig. 6:**
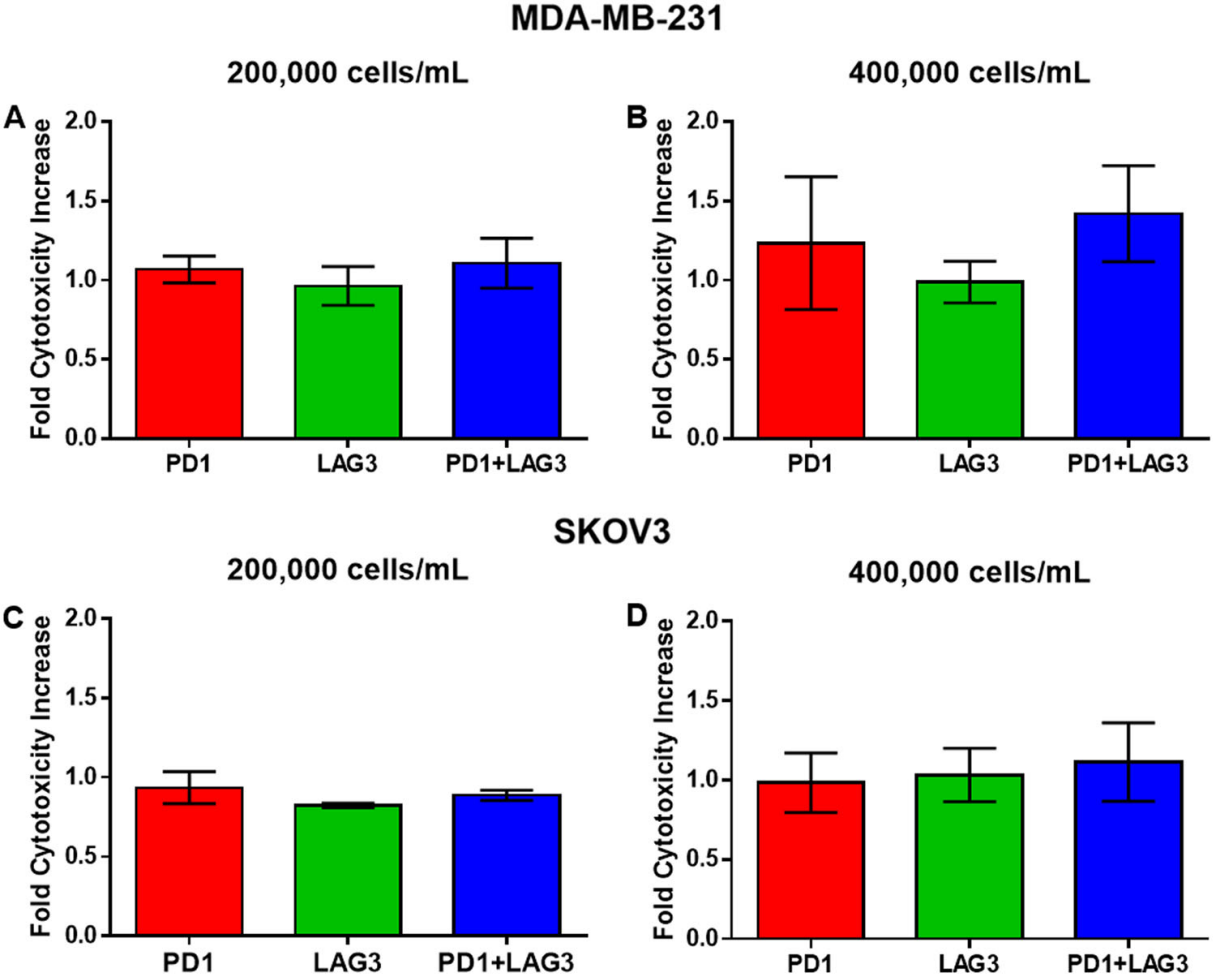
Cytotox 96 plate 24-h co-encapsulations of CD8^+^ T cells and target cancer cells endpoint readings normalized to control. **A** Plates seeded with 200,000 MDA-MB-231 breast cancer cells per mL concentration. **B** Plates seeded with 400,000 MDA-MB-231 cells per well target concentration. **C** Plates seeded with 200,000 SKOV3 ovarian cancer cells per well. **D** Plates seeded with 400,000 SKOV3 cells per well. All conditions and controls were in triplicate wells. Error bars represent the standard error of the mean.

To compare the effects of TSR-042 and TSR-033 observed in our droplet-based single-cell assay to a standard in vitro assay, we performed 24-h co-cultures of MDA-MB-231 and SKOV3 cells with CD8^+^ T cells subject to control conditions and to checkpoint inhibitors alone or in combination. Cytotoxicity of each condition was determined via LDH release with the CytoTox 96 colorimetric assay. Cells were cultured at a 1:1 ratio to match droplet data, and two different concentrations of cells were tested; 200,000 and 400,000 cells/mL. These results show similar MDA-MB-231 death for each treatment and control condition for the 200,000 cell/mL concentration (Fig. 6A). For the 400,000 cell/mL concentration the combination treatment resulted in a slight increase in cytotoxicity to control, however, this difference was not enough to be significant (*p* = 0.31) (Fig. 6B). For SKOV3, neither concentration of cells produced a significant increase in cytotoxicity for any of the treatment conditions versus untreated control (Fig. 6C, D).

## Discussion

In this study, we sought to characterize the effects of TSR-042 and TSR-033 on CD8^+^ T cells at the single-cell level. To estimate how these antibodies might interact with our target cell lines, we tested for the expression of PD-L1, the primary ligand for PD1, and HLA-DP, DQ, DR, the primary ligand for LAG3. These results match previous findings of MDA-MB-231 cells having a high expression of PD-L1^12^, and slightly more variable but relatively high expression of HLA-DP, DQ, DR^28,29^ Also in adherence with literature, SKOV3 displayed a more moderate expression of PDL1^30^. HLA-DP, DQ, DR expression was negligible in SKOV3, however, there are several other potential ligands theorized to interact with LAG3 not tested here^31–33^. The expression of PD1 and LAG3 we observed also correlate to previous studies of checkpoint receptor expression on CD8^+^ T cells. The expression of these receptors has been shown to be highly variable and to correlate significantly with T-cell exhaustion. Collectively, these results show that out activation protocol generates adequate exhaustion to induce checkpoint receptor expression, and suggests a high potential for checkpoint interactions between these tumor cells and the CD8^+^ T cells in droplet co-encapsulations, as well as in a tumor microenvironment. The different checkpoint ligand expression between SKOV3 and MDA-MB-231 also creates an interesting comparison for the efficacy of TSR-042 and TSR-033 with cell lines.

Testing the impact of TSR-042 and TSR-033 on the activation of CD8^+^ T cells by dendritic cells revealed that blocking the LAG3 receptor enhances Ca^2+^ stimulation in CD8^+^ T cells most effectively out of these two checkpoint pathways. This may be related to previous findings of LAG3 inhibitions effects on CD8^+^ T-cell activity, despite the limited expression of LAG3 in comparison to PD1^10^. Indeed, high expression of LAG3 ligands on DCs, including HLA-DR and LSECTin has been found, in comparison to the ligands for PD1^10^. Calcium signaling is known to have a variety of important roles in T-cell activity and maturation. These results suggest that TSR-033 inhibition of LAG3 has considerable potential to increase the activity of exhausted CD8^+^ T cells, which may increase their cytotoxic potential in the tumor microenvironment. The lack of statistical difference between PD1 inhibition and control, and between the combination treatment and LAG3 suggest PD1 inhibition is not significantly affecting the activation of CD8^+^ T cells by DCs.

In the single-cell encapsulations of CD8^+^ T cells with MDA-MB-231, PD1 inhibition caused a modest increase in cytotoxicity and LAG3 inhibition had little to no increase in cytotoxicity of T cells. The combination of both resulted in gradually increasing efficacy over the course of 24-h co-encapsulations. This may be due to synergistic effects of blocking the PD1 and LAG3 at mediating the T-cell exhaustion induced by the weeklong activation protocol, allowing the combination-treated T cells to continue killing longer than control T cells. For the encapsulations with SKOV3, we observed no significant differences between the treatment conditions and lesser increases in cytotoxicity of each condition versus control as compared to MDA-MB-231. This indicates that these checkpoint pathways play less of a role in SKOV3 resistance to CD8^+^ T cytotoxicity. In the CytoTox 96 assay, no significant differences were observed across conditions for either target cell, exemplifying the lack of sensitivity of traditional in vitro cytotoxicity assays.

In addition to cytotoxicity data, this platform also enabled observation of synaptic contact between cells. Both SKOV3 and MDA-MB-231 tended to have the lowest average contact duration with CD8^+^ T cells in the combination treatment. The mechanism responsible for this variation in contact duration is uncertain, however, it may be due to the localization of antibody-bound PD1 and LAG3 receptors to the site of synaptic contact, destabilizing the binding of T-Cell Receptor (TCR) to MHC-I^6^. The destabilization of TCR–MHC-I binding may also be responsible for the increase in CD8^+^ T-cell contact frequency with MDA-MB-231 during LAG3 inhibition in Fig. 5D however why LAG3 inhibition results in an increase in contact frequency while PD1 blocking and combination treatment do not for MDA-MB-231 is unclear. For SKOV3 co-encapsulations, the reduced number of contacts observed in the treatment conditions is most likely a result of the reduced time required for CD8^+^ T cells to kill, as observed in the time-to-death figure (Fig. 4F).

For MDA-MB-231, similar average contact times were observed for all conditions where target cells were killed (Fig. 5C), suggesting checkpoint inhibition did not significantly impact the duration of time needed for CD8^+^ T cells to kill MDA-MB-231 breast cancer cells. Conversely, the contact time was significantly shorter for SKOV3 cells killed by CD8^+^ T cells in the presence of both antibodies. This suggests that, while not significantly increasing the cytotoxicity towards SKOV3 cells in these experiments, TSR-033 and TSR-042 decreased the time needed for CD8^+^ T cells to kill when combined. The effects of this are reflected in the multi-target killing (Fig. 4E) and time-of-death (Fig. 4F) for SKOV3. These figures indicate that, while the combination of these antibodies did not appear to increase the overall cell death, they do result in a change in the kinetics of CD8^+^ T-cell cytotoxicity against SKOV3.

## Conclusion

We here applied an advanced droplet-based microfluidic assay to the evaluation of two novel checkpoint inhibitors, TSR-042-targeting PD1 and TSR-033-targeting LAG3, designed for cancer immunotherapy. Observations of the interactions between cells in the presence of these antibodies were not possible with previously used in vitro tests, which typically aimed at indirect observation of CD8^+^ T cells activation rather than measuring actual tumor-killing. These interactions included characterization of contact dynamics and real-time monitoring of cytotoxicity, as well as a controlled means for evaluating serial-killing or multiple-killing events by the same effector cell. The assay revealed novel insights and data at a single-cell resolution on the impact of PD1 and LAG3 inhibition (both separately and in combination) on CD8^+^ T cells activation by exposure to antigen-presenting cells such as dendritic cells and on CD8^+^ T cells tumor-killing function. Overall, these data revealed distinctly different responses between the two target cancer cell lines to CD8^+^ T cells treated with TSR-033 and/or TSR-042 combination. None of these differences were observable in the traditional in vitro assay we utilized for comparison. These results demonstrate that, while promising, the efficacy of TSR-033 and TSR-042 as combination therapy is greatly affected by the targeted cancer type. Advanced in vitro platforms such as this device may enhance the predictability of treatment efficacy with this combination and other immunotherapies.

## Supplementary information

Supplementary Methods
