# Supplementary material for: Quantifying the efficacy of checkpoint inhibitors on CD8+ cytotoxic T cells for immunotherapeutic applications via single-cell interaction
Source: Cell Death Dis. 2020 Nov 13;11(11):979. doi: 10.1038/s41419-020-03173-7 (PMC7666200; doi:10.1038/s41419-020-03173-7)
Supplement: Supplementary file 1 — Supplementary Methods [file 41419_2020_3173_MOESM1_ESM.docx]

**Imaging and Cell Analysis.**

In all experiments, cells were quickly injected in the droplet-based microfluidic device and approximately 4000 droplets were trapped in the array for analyses. For each microfluidic device approximately 100 droplets containing cells of interest (T-cell:tumor cell or T-cell: dendritc cell) were selected for analysis. Droplet imaging was performed using a Zeiss Axio Observer.Z1 Microscope (Zeiss, Germany) equipped with a Hamamatsu digital camera C10600 Orca-R2. Microfluidic devices were maintained in the microscope environmental chamber at standard conditions for the duration of the experiment

For Calcium dynamics imagining, T Cells were labeled with Fluo-4 NW Calcium Assay dye (Life Technologies, Carlsbad, CA) at 1X concentration for 30 to 45 minutes at 37°C. T cells and DCs were encapsulated into droplets in the microfluidic devices. Droplets utilized for analysis all contained 1 to 2 cells of each type. Droplets were imaged every 1 minute for approximately 4.5 hours. T cell fluorescence intensity was measured to interpret activation events using the integrated density function of ImageJ and normalizing this data to the initial time point. The normalized data was graphed and analyzed to determine activation peaks.

For cytotoxicity, cell viability in droplets was determined by incorporating the Live/Dead Viability/Cytotoxicity assay reagents in droplets (Life Technologies, Carlsbad, CA). Calcein AM, the live cell indicator, was assessed by time-lapse microscopy at excitation/emission: 494/517 nm. Ethidium homodimer (EthD-1), the dead cell indicator, was read at 528/617 nm. The proportion of live cells was calculated as a ratio of the number of live cells to the total number of cells as expressed as ‘percentage viability’. Typically, only droplets with 1 target and 1 effector cell were recorded. For observations of serial killing in droplets, higher concentrations of cancer cells were loaded and droplets with 2 target cells and 1 effector cell were selected for observation. In T cell viability experiments (T cells alone without cancer cells), the T cells were labeled with 500 ng/mL Calcein AM for 30 minutes at 37°C prior to the experiment. In cytotoxicity experiments (paired T-cells and cancer cells) tumor cells were labeled with Calcein AM. For all viability/cytotoxicity experiments, EthD-1 was included in the droplets at 2 µM concentration to identify dead cancer cells and T cells. Experiments were conducted over the course of 24 hours, with frame acquisition every 15 minutes. Cytotoxicity and immunological synapse duration between T cells and cancer cells were assessed through visual analysis using the Zen Blue software from Zeiss Microscopy. Cell death was determined via visual analysis based on the loss of green fluorescence, acquisition of red fluorescence and characteristic changes in cellular morphology. Cells dead at onset of experiment were not included in calculations. Statistical comparisons of cytotoxicity were calculated using the Welch’s t-test, and contact duration was compared using the Mann-Whitney U test.

**Standard In Vitro Cytotoxicity.**

96 well co-cultures of SKOV3 or MDA-MB-231 cancer cells and CD8+ T cells were conducted as a standard in vitro cytotoxicity test to be used as comparison. The Promega Cytotox 96 kit and protocol were utilized for these experiments, which detects lactate dehydrogenase (LDH) released from cells upon lysis. T cells were incubated with checkpoint inhibitor antibodies at concentrations of 30 µg/mL for 2 hours prior to and during incubation. Control cells were treated with human IgG4 isotype control. Cells were seeded at 1:1 Effector:Target concentrations at both 20,000 and 40,000 target cells per well. Plates were incubated at 37° C and 5% CO_2_ for 24 hours. LDH was labeled following the kit protocol, and measured using a BioTek Synergy HT 96 well plate reader at 490 nm absorbance. Total target cell death was calculated with the following equation:

$\frac{Experimental-Effector Spontaneous LDH Release}{Target Max LDH Release}$ (Eqn. 1)

Since total target cell death is recorded in the droplets, this provides the most comparable value to the cytotoxicity observed in single cell encapsulations.

**Flow cytometry.**

Surface expression of checkpoint receptors on T cells and checkpoint ligand expression on cancer cells was determined using fluorescent antibody labeling and flow cytometry. The MDA-MB-231 breast cancer cell line and SKOV3 ovarian cancer cell line were tested for the surface expression of PDL1 (a PD1 ligand) and HLA-DP,DQ,DR (LAG3 ligands) using PE-labeled anti-human mouse antibodies and isotype controls (Biolegend, San Diego, CA). Expression of checkpoint receptors on T cells was determined using PE-labeled anti-human mouse antibodies for PD1 and PE-cy7-labeled anti-human mouse antibodies for LAG3 (BD Biosciences, Woburn, MA). Viability of cells was determined using Calcein AM. 10 repeats were performed for the receptor analysis on T cells and 2 repeats were conducted for ligand analysis on cancer cells, with each approximately 30,000 cells observed per repeat. All experiments were run on a BD FACSCalibur (BD Biosciences, Woburn, MA). Results and statistical analysis were performed using FlowJo software (FlowJo, LLC, Ashland, OR).
